# Prevalence of rheumatic and musculoskeletal diseases (RMDs) in nursing home residents: a systematic literature review

**DOI:** 10.1007/s41999-024-01067-x

**Published:** 2024-09-25

**Authors:** Shennah Austen, Iris Kamps, Annelies E. R. C. H. Boonen, Jos M. G. A. Schols, Marloes G. B. van Onna

**Affiliations:** 1 Cicero Zorggroep, P.O. Box 149, Zuid-Limburg, 6440 AC Brunssum The Netherlands; 2 Department of Health Services Research, Maastricht University, Maastricht, The Netherlands; 3 Division of Rheumatology, Department of Internal Medicine, Maastricht University Medical Center, Maastricht, The Netherlands; 4 School for Public Health and Primary Care (CAPHRI), Maastricht University, Maastricht, The Netherlands

**Keywords:** Nursing home, Rheumatic and musculoskeletal diseases, Prevalence

## Abstract

**Purpose:**

The objective of this systematic literature review was to: (1) estimate the prevalence of (symptoms of) rheumatic and musculoskeletal diseases (RMDs) and (2) explore how (symptoms of) RMDs are identified and documented in studies among nursing home residents.

**Methods:**

Prevalence data of (symptoms of) RMDs in permanently admitted nursing home residents ≥ 60 years were included. Data extraction, data synthesis and risk of bias assessment were performed by two reviewers independently. Included studies were categorized based on case ascertainment and case definition comprising: (location of) musculoskeletal pain, general terms for RMDs or a specific type of RMD. Results were summarized descriptively.

**Results:**

Out of 6900 records, 53 studies were included. Case ascertainment comprised databases (*n* = 5), physical examination (*n* = 1), self-report questionnaires (*n* = 14), review of medical charts (*n* = 23) and self-report questionnaires combined with review of medical charts (*n* = 10). Prevalence ranged between 0.9 and 77.0% for (localized) musculoskeletal pain (*n* = 19) and between 0.6 and 67.5% for RMDs in general (*n* = 39). Prevalence rates of specific type of RMDs ranged between 0.7 and 47.5% for gout, between 3.3 and 11.0% for rheumatoid arthritis and between 2.8 and 75.4% for osteo-arthritis (*n* = 14). Heterogeneity with regard to documentation of (symptoms of) RMDs in medical data of nursing home residents was high.

**Conclusion:**

The overall prevalence of (symptoms of) RMDs varied to a great extent. This was mainly due to large heterogeneity in documentation of (symptoms of) RMDs. Establishing agreement on a useful and practical classification may ultimately increase identification of RMDs in the nursing home setting.

**Supplementary Information:**

The online version contains supplementary material available at 10.1007/s41999-024-01067-x.

## Key summary points

**Aim:**

To (1) estimate the prevalence of (symptoms of) rheumatic and musculoskeletal diseases (RMDs) and (2) explore how (symptoms of) RMDs are identified and documented in studies among nursing home residents.

**Findings:**

The prevalence of RMDs in nursing home residents varied to a great extent due to large heterogeneity in documentation of (symptoms of) RMDs.

**Message:**

Establishing agreement on a useful and practical classification may ultimately increase identification of RMDs in the nursing home setting.

**Supplementary Information:**

The online version contains supplementary material available at 10.1007/s41999-024-01067-x.

## Introduction

Rheumatic and musculoskeletal diseases (RMDs) are the most common cause of chronic pain and physical disability worldwide [1–4]. There are more than 200 RMDs that generally affect the joints, but also may affect muscles, tendons and internal organs [1, 2, 4]. RMD-related pain is usually associated with limitations in physical function and at times also with limited range of motion [1, 2]. In some RMDs, there are local signs of inflammation, such as joint swelling, redness and warmth in the affected areas [2, 4]. Osteo-arthritis, non-specific low back pain and pain due to previous fractures are common RMDs in community-dwelling older people [1, 3, 5, 6]. According to the World Health Organisation (WHO) 10% of men and 20% of women over the age of 60 have symptomatic osteo-arthritis [5].

Both changes in symptom presentation and presence of co-morbidities in older people can pose diagnostic problems and contribute to both over- and undertreatment of RMDs. This is especially the case in nursing home residents [7–11]. Nursing home residents often have chronic somatic diseases or progressive dementia and need complex continuing care in multiple domains [12, 13]. Frequent co-morbidities of nursing home residents include cerebrovascular disease, other neurological diseases (e.g., Parkinson disease), malignancies and depression which all may be associated with (musculoskeletal) pain [10–12, 14, 15]. In addition, these concomitant diseases and cognitive impairment may ultimately mask, mimic or overshadow RMD-related symptoms in nursing home residents [7, 9, 16, 17].

Chronic pain in nursing home residents is associated with anxiety, depression, loneliness, social isolation and frequent falls [8, 9, 14]. On top of this, RMDs in older persons are characterized by a cycle of disuse and inactivity which leads to further reduction of function due to sarcopenia [5, 6, 8]. RMD-related symptoms such as pain and stiffness are often wrongly perceived by patients and health-care workers as being a normal consequence of aging [7, 9]. Early recognition and tailored treatment of RMDs may prevent further loss of mobility and increasing care dependency, improve quality of life and the quality of medical care of nursing home residents.

Insight into the prevalence of RMDs and RMD-related symptoms in nursing home residents is currently low. Studies that describe prevalence rates of (symptoms of) RMDs in nursing home residents are heterogeneous with regard to selection of patients, outcome (e.g., pain with or without description of anatomical location or type of RMD) and source of outcome (e.g., medical chart or database) [8, 14, 18]. A summary of the literature could help to provide insight in prevalence rates of (symptoms of) RMDs by organizing scattered information and identifying causes of clinical or methodological variation. The aim of this systematic literature review (SLR) was, therefore, to (1) estimate the prevalence of (symptoms of) RMDs in studies among nursing home residents and (2) explore how (symptoms of) RMDS are identified and documented.

## Methods

This SLR was performed in accordance with the preferred reporting items for systematic reviews and meta-analyses (PRISMA) guidelines [19]. The protocol was registered at the PROSPERO International Prospective Register of Systematic Reviews (CRD42022310221).

### Search strategy

The search was conducted in MEDLINE/Pubmed, EMBASE, Cumulative Index to Nursing and Allied Health Literature (CINAHL) and Web of Science from date of inception to August 1st, 2024. We used a combination of free text words and controlled vocabulary terms (e.g., MeSH terms) in relation to RMDs, nursing home residents and prevalence or epidemiology. The detailed search strategy is outlined in Supplementary Material S1. The reference list of all included studies was hand-searched to identify additional studies of interest. Studies published in other languages than English, German, French, Spanish, Portuguese and Dutch were excluded. Letters to the editor, case reports, reviews and editorials were also excluded.

### Study selection

Studies were included if: (1) permanently admitted nursing home residents were the target population, (2) residents were 60 years and older, and (3) the study provided data on the epidemiology of RMDs. For the definition of nursing home residents, we used the stated consensus of the International Association of Gerontology and Geriatrics (IAGG) and the American Medical Directors Association (AMDA) foundation [13]. Studies were excluded if they described a study population with: (1) exclusively acute traumatic fractures and musculoskeletal injuries, (2) exclusively osteoporosis or (3) short-term or short-stay nursing home residents, patients staying in geriatric rehabilitation care units or exclusively community-dwelling persons. A two-stage screening process was performed by two authors (SA and IK). First, title and abstracts were screened for eligibility. In the second step, the full-text articles of the selected titles were evaluated. Disagreements between the first two assessors were resolved through consensus or involvement of a third assessor (MO).

### Data extraction

Data extraction was performed independently by two reviewers using a standardized data extraction form. Any disagreement was resolved through consensus or by consulting a third reviewer (MO). Data extraction included: study identification (first author, publication year), study characteristics (study design, country, inclusion period, setting, inclusion criteria, sample size), patient characteristics (age, gender, reasons for nursing home admission, medical co-morbidities), prevalence data related to frequency, anatomical location of pain, type of RMDs and when available the criteria used to verify the diagnosis of the RMDs (case ascertainment).

### Risk of *bias* assessment

The methodological quality of the included studies was assessed by two independent reviewers (SA and IK) using the critical appraisal tool for assessing the quality of cross-sectional studies (AXIS) [20]. Disagreements were resolved through consensus or involvement of an adjudicator (MO). The AXIS tool consists of a 20-item questionnaire with a dichotomic scale. Key areas in the AXIS tool include study design, sample size justification, target population, sampling frame, sample selection and measurement of validity and reliability. The scores were categorized into quartiles: > 15 AXIS criteria met (high quality), 10‐15 AXIS criteria met, 5‐9 AXIS criteria met, and ≤ 4 AXIS criteria met (low quality).

### Data synthesis and analysis

Due to the large clinical and methodological heterogeneity of the included studies, we chose to summarize the results descriptively. First, studies were categorized based on case ascertainment, i.e., database study, performing a physical examination, self-reported diagnosis (or symptoms) and review of the medical chart. Second, studies were categorized based on case definition. The case definition was divided into three categories comprising (1) (location of) musculoskeletal pain (e.g., “pain in arm or back”), (2) general terms for RMDs (e.g., “arthritis”) or (3) a specific type of RMD (e.g., “osteo-arthritis”).

## Results

### Study selection

**Fig. 1:**
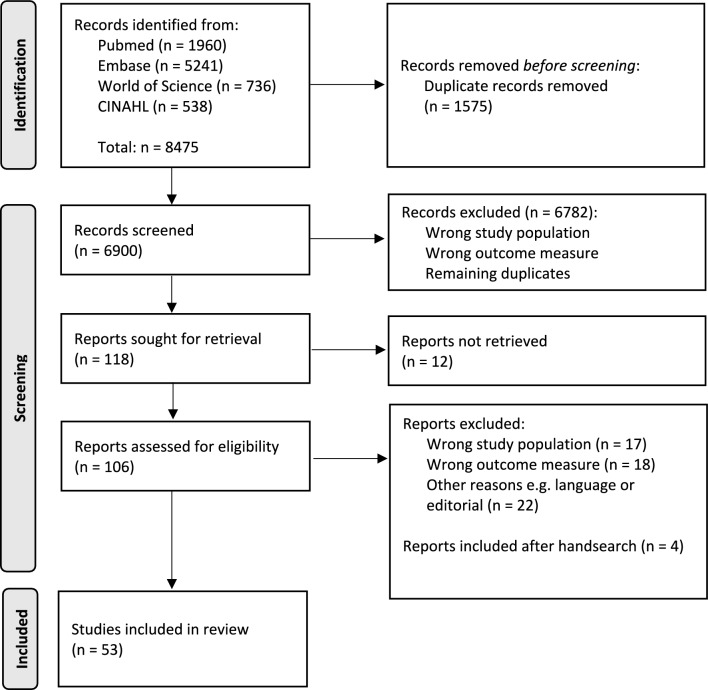
PRISMA flow diagram of the literature search

The literature search provided 6900 non-duplicated articles. After screening the titles and abstracts for eligibility, 118 studies remained for full paper review. For 12 studies, no full text could be retrieved. After full-text reading, 57 articles did not meet the inclusion criteria (17 articles described a wrong study population, 18 articles described wrong outcome measurements, and 22 articles were excluded for other reasons such as a case report) and 49 studies were included. An additional four studies were included after handsearching and checking reference lists (see Fig. 1) [9, 10, 14, 15, 18, 21–68].

### Population and case ascertainment

#### Study characteristics

The 53 included studies were performed between 1989 and 2024 (see Table 1). Studies were mainly carried out in North America (*n* = 13; 24.5%), Europe (*n* = 15; 28.3%) and Australia (*n* = 6; 11.3%). The number of study participants ranged from 21 to 160,9000. Approximately, 62.9% (range 0–100%) of the study population was female. The mean age of the studied populations varied between 62.4 ± 13.9 and 88.4 ± 5.9 years with a mean across studies of 81.2 years (data of mean age available in 41 studies). All studies had a cross-sectional study design. Case ascertainment was based on large databases in five studies (9.4%) [45, 47, 48, 54, 62], performance of a physical examination in one study (1.9%) [50], self-report questionnaires in 14 studies (26.4%) [14, 25–28, 32, 38, 39, 44, 46, 49, 58, 61, 65] and review of medical charts in 23 studies (43.4%) [9, 15, 18, 21, 22, 24, 29–31, 34–37, 40–42, 51–53, 55, 63, 67, 68]. In ten studies (18.9%), the medical chart was reviewed in combination with a self-report questionnaire (see Table 2) [10, 23, 33, 43, 56, 57, 59, 60, 64, 66].

**Table 1:** Characteristics of included studies

Author, publication year	Country, *n*, case ascertainment	Mean age ± SD/range (years)	Mean woman (%)	Dementia (%)	Prevalence (location) of musculoskeletal pain (%)	Prevalence RMDs in general (%)	Prevalence specific type of RMD (%)
Abell, 2004 [21]	USA, 1,609,000, review medical chart	NA	81.2%	NA	NA	Arthritis; 19.0%	NA
Achterberg, 2007 [22]	The Netherlands, 562, review medical chart	78.5 ± 10.5	64.6%	47.3%	Back; 12.3% Bone; 5.3% Thorax; 1.2% Head; 5.0% Pelvis; 2.3% Hip; 10.3% Joint; 11.4% Arms/ legs; 14.2% Soft tissue; 8.5%	NA	OA; 13.3%
Alaba, 2009 [23]	Spain, 86, review medical chart and self-report questionnaire	84.9 ± 8.1	NA	46.0%	Shoulder; 7.5% Back; 37.7% Knees; 28.3% Feet; 16.9%	NA	NA
Al-Momani, 2016 [24]	Jordan, 221, review medical chart	62.4 ± 13.9	45.2%	47.5% (cognitive impaired, advanced dementia excluded)	NA	Arthritis; 9.5% Joint disease; 17.6%	NA
Albertsen, 2021 [25]	Greenland, 244, self-report questionnaire	77.0	62.3%	29.5%	NA	Musculoskeletal disease; 25.8%	NA
Algameel, 2020 [26]	Egypt, 63, self-report questionnaire	NA	66.6%	0.0%	NA	Arthritis; 26.9%	NA
Altiparmak, 2011 [27]	Turkey, 106, self-report questionnaire	NA	39.6%	0.0%	NA	Musculoskeletal disorders; 2.8%	NA
Bekhet, 2014 [28]	USA, 314, self-report questionnaire	83.0	79.6%	0.0%	NA	Arthritis; 23.0%	NA
Black, 2006 [29]	USA, 123, review medical chart	81.5 ± 7.1	55.3%	100.0% (all advanced dementia)	NA	Musculoskeletal disease; 61.0% Arthritis; 49.6%	NA
Boerlage, 2008 [10]	The Netherlands, 157, review medical chart and self-report questionnaire	88.0	NA	0.0%	Legs; 32.0% Lower back: 27.0% Shoulders/ arms; 13.0%	Diseases of the musculoskeletal system and connective tissue; 35.1%	NA
Chen, 2023 [64]	China, 387, review medical chart and self- report questionnaire	82.6 ± 7.4	47.8%	NA	NA	Orthopedic diseases; 27.9%	NA
Cook, 1998 [30]	Canada, 21, review medical chart	77.6	61.9%	0.0%	NA	Unspecified arthritis; 7.0%	OA; 36.0% RA; 11.0%
D’Astolfo, 2006 [31]	Canada, 140, review medical chart	83.7	69.3%	96.4%	Musculoskeletal pain; 40.0% Head; 6.0% Neck; 2.0% Back; 21.0% Extremity; 33.0% Unidentified; 38.0%	NA	OA; 25.0%
Damian, 2004 [32]	Spain, 800, self-report questionnaire	83.4 ± 7.3	75.0%	44.8%	Joint pain; 40.0%	Arthritis and severe OA; 34.0%	NA
Decker, 2009 [33]	USA, 215, review medical chart and self-report questionnaire	86.4 ± 7.3	75.8%	51.6%	Musculoskeletal pain; 77.0%	Degenerative joint disease or osteo-arthritis or arthropathy; 66.5%	OA; 47.5%
Ferrell, 1990 [34]	USA, 92, review medical chart	88.4 ± 5.9	83.0%	37.0% (cognitive impaired, advanced dementia excluded)	Low back; 40.0% Previous fractures; 14.0% Knee; 9.0% Shoulder; 8.0% Foot; 8.0% Hip; 6.0% Neck; 6.0%	Arthritis; 45.0%	NA
Finne-Soveri, 2000 [35]	Denmark, Finland, Sweden, Iceland, 6487, review medical chart	83.2	74.4%	33.0%	Pain caused by arthritis; 38.6%	NA	NA
Fisher, 2002 [36]	USA, 57, review medical chart	82.2 ± 8.0	NA	92.9%	NA	Musculoskeletal diseases; 38.6%	OA; 21.1%
Gill, 2022 [37]	Australia, 490,325, review medical chart	8.4 ± 7.2	NA	NA	NA	Musculoskeletal conditions; 40.2% Arthritis; 36.5%	NA
Gerber, 2016 [38]	South-Africa, 104, self-report questionnaire	77.0	72.1%	25.7%	NA	Joint disorders; 46.2%	NA
Grimby, 1999 [39]	Norway, 1800, self-report questionnaire	NA	76.2%	NA	Musculoskeletal pain; 64.8% Back; 35.3% Joints; 30.4% Shoulders; 43.1%	NA	NA
Guccione, 1989 [40]	USA, 629, review medical chart	NA	NA	45.4%	NA	Arthritis; 23.0%	NA
Hillen, 2017 [41]	Australia, 167,543, review medical chart	NA	69.3%	48.0%	NA	Musculoskeletal condition; 19.4% Arthritis; 13.4%	NA
Hsieh, 2021 [42]	Taiwan, 4119, review medical chart	77.9 ± 13.2	52.5%	98.1%	Low back pain; 6.6%	Orthopedic disease; 7.4% Other musculoskeletal disorders; 9.7% Polymyalgia; 0.6%	Gout; 0.7%
Jerez-Roig, 2016 [43]	Brazil, 144, review medical chart and self-report questionnaire	79.4 ± 8.2	79.2%	19.5%	NA	Rheumatic diseases; 11.8%	NA
Kalideen, 2022 [44]	South-Africa, 102, self-report questionnaire	78.9 ± 8.1	74.5%	8.8% (cognitive impaired, advanced dementia excluded)	NA	Arthritis; 38.2%	NA
Karmel, 2012 [45]	Australia, 33,300, database	80.7	59.0%	18.4%	NA	Musculoskeletal disease; 12.6% Arthritis; 8.7% Other musculoskeletal disease; 2.0%	NA
Laikhuram, 2024 [65]	India, 422, self-report questionnaire	72.0 ± 6.5	77.9%	NA	NA	Musculoskeletal diseases; 17.1%	NA
Lapane, 2012 [46]	USA, 9952, self-report questionnaire	NA	65.2%	55.0%	Back; 4.1% Joint; 4.4% Soft tissue; 1.7% Bone; 0.9% Other; 8.0%	NA	NA
Lind, 2020 [47]	Australia, 9436, database	NA	67.2%	58.0%	NA	Arthritis; 60.7%	NA
Luque Ramos, 2017 [48]	Germany, 75,697, database	83.9 ± 6.6	77.7%	NA	NA	NA	RA; 3.3%
Marques, 2015 [49]	Portugal, 329, self-report questionnaire	NA	79.6%	90.6%	NA	Osteo-articular problems; 22.2%	NA
Martinez, 2011 [50]	Spain, 171, physical examination	81.3	100.0%	NA	NA	Foot conditions; 26.7%	NA
Monroe, 2011 [51]	USA, 92, review medical chart	81.0	80.4%	84.0%	NA	Degenerative joint disease; 57.6%	NA
Moore, 2012 [52]	USA, 11,788, review medical chart	84.0 ± 8.0	74.5%	51.0%	NA	Arthritis; 33%	NA
Myrenget, 2023 [66]	Norway, 262, review medical chart and self-report questionnaire	88.0	71.8%	28.2%	Chronic primary musculoskeletal pain; 32.5% Chronic secondary musculoskeletal pain; 31.3% Pain from OA; 24.0% Secondary musculoskeletal pain from spine; 9.2% Musculoskeletal pain from inflammatory joint; 5.0% Secondary musculoskeletal pain from upper limb; 4.6% Cervicalgia; 3.8% Primary musculoskeletal pain from neck; 4.6% Pain in lower leg; 6.1% Pain in hip; 6.1% Primary musculoskeletal pain lower limb; 14.1% Primary musculoskeletal pain upper limb; 19.8% Non-specific shoulder pain; 16.0% Pain in thoracic spine; 4.2% Low back pain; 18.7% Nonspecific pain from back; 23.3%	Impingement syndrome shoulder; 1.1%	OA knee; 11.1% OA hip; 10.3%
Ng, 2020 [53]	Canada, 19,477, review medical chart	NA	65.7%	54.1%	NA	NA	OA; 75.4% RA; 3.6%
Nguyen, 2020 [54]	Australia, 11,548, database	NA	33.7%	46.1%	NA	NA	Gout; 10.7%
Peng, 2009 [55]	Taiwan, 574, review medical chart	80.9 ± 5.4	0.0%	20.2%	Lower back; 40.5% Bone; 5.2% Hip joint; 5.2% Joints; 29.4% Soft tissue; 5.2%	NA	NA
Proctor, 2001 [56]	Canada, 3195, review medical chart and self-report questionnaire	84.8	69.0%	71.5%	NA	Arthritis; 16.1%	NA
v Rensbergen, 2010 [15]	Belgium, 691, review medical chart	84.4 ± 8.3	78.0%	24.2%	NA	Musculoskeletal disease; 14.0%	OA; 26.0%
Sawyer, 2007 [9]	USA, 26,110, review medical chart	83.0 ± 7.9	75.1%	49.7%	NA	Musculoskeletal diagnosis; 52.6%	NA
Sigurdardottir, 2018 [57]	Iceland, 5242, review medical chart and self-report questionnaire	82.5 ± 8.4	58.4%	25.5%	NA	Arthritis; 1.5%	NA
Takai, 2013 [58]	Japan, 5219, self-report questionnaire	NA	NA	NA	NA	Arthritis; 53.2%	NA
Tansug, 2021 [14]	Turkey, 73, self-report questionnaire	75.9 ± 8.1	37.0%	NA (Advanced dementia excluded)	Musculoskeletal pain; 57.5%	NA	NA
Torvik, 2009 [59]	Norway, 214, review medical chart and self-report questionnaire	86.0 ± 6.5	71.5%	41.0%	NA	Myalgia and arthritis; 13.0%	NA
Tsai, 2004 [60]	Taiwan, 150, review medical chart and self-report questionnaire	80.7 ± 7.4	58.7%	3.3% (Advanced dementia excluded)	Knee; 27.6% Lower back; 24.5% Hips; 18.4%	NA	OA; 13.4%
Tse, 2004 [61]	China, 44, self-report questionnaire	NA	84.1%	0.0%	Whole body; 18.2% Knee; 11.4% Ankle; 11.8% Back; 15.9% Legs; 15.9% Back and knee; 6.8%	Musculoskeletal problems; 67.5%	NA
Veal, 2019 [62]	Australia, 382, database	85.1	65.3%	NA	Musculoskeletal pain, lower extremities; 50.0% Back or neck pain; 35.1% Musculoskeletal pain, upper extremities; 29.3%	NA	NA
Vetrano, 2022 [67]	Italy, 4131, review medical chart	84.3 ± 8.4	70.3%	61.7%	NA	Other musculoskeletal disease; 5.6%	OA; 2.8%
Xie, 2023 [68]	China, 202, review medical chart	79.1 ± 9.1	43.1%	NA	NA	Bone and joint disease; 13.3%	NA
Zanocchi, 2008 [63]	Italy, 105, review medical chart	82.2 ± 9.0	70.5%	100.0%	Knee; 19.5% Hip; 16.5% Back; 11.5%	Osteo-articular system problem; 57.1%	NA
Zarowitz, 2013 [18]	USA, 138,724, review medical chart	NA	63.2%	NA	Back; 14.7% Bone; 4.4% Hip; 6.3% Joint (other than hip); 20.6%	NA	Gout; 1.8%

*RA* rheumatoid arthritis, *OA* osteo-arthritis, *NA* not applicable

**Table 2:** Summary of prevalence rates of included studies

Case ascertainment (study design)	Number of studies (*n*)	Prevalence musculoskeletal pain (range; %)	Prevalence RMDs in general (range; %)	Prevalence specific type of RMD (range; %)
Databases	5	31.8% (*n* = 1)	2.0–60.7% (*n* = 2; median 10.7%)	RA; 3.3% (*n* = 1) Gout; 10.7% (*n* = 1)
Physical examination	1	NA	26.7% (*n* = 1)	NA
Self-report questionnaire	14	0.9–64.8% (*n* = 5; median 19.2%)	2.8–67.5% (*n* = 11; median 26.9%)	NA
Medical chart	23	1.2–40.5% (*n* = 8; median 9.7%)	0.6–61.0% (*n* = 18; median 26.0%)	RA; 3.6–11.0% (*n* = 2) Gout; 0.7–1.8% (*n* = 2) OA; 3.6–75.4% (*n* = 6; median 28.6%)
Self-report questionnaire and medical chart	10	7.5–77.0% (*n* = 5; median 24.0%)	1.1–66.5% (*n* = 7; median 13.0%)	OA; 10.3–47.5% (*n* = 3; median 13.4%)

### Prevalence by case definition

#### Musculoskeletal pain in general or by anatomic location

Nineteen studies described an overall prevalence rate for the category (location of) musculoskeletal pain ranging between 0.9% [46] and 77.0% [33] with a median of 13.5%. Seven studies used a general term such as “musculoskeletal pain” without further defining where exactly the pain was localized (prevalence rate: 4.6–77.0%; median 36.3%) [14, 31–33, 35, 39, 66]. Three studies documented “joint pain” (prevalence: 40.0%), “musculoskeletal pain from inflammatory joint” (prevalence: 5.0%) or “pain caused by arthritis” (prevalence: 38.6%), without specifying cause or location of arthritis [32, 35, 66]. Fifteen studies described “pain in a body part or structure” with prevalence rates varying from 0.9% (“pain bone”) [46] to 43.1% (“pain shoulder”) [39] with a median of 11.8%.

One study used a database to generate a prevalence rate of musculoskeletal pain (upper extremities: 29.3%, back or neck pain: 35.1%, lower extremities: 50.0%) [62]. Five studies used a self-report questionnaire and found prevalence rates between 0.9% [46] and 64.8% [39]. Prevalence rates varying between 1.2% [22] and 40.5% [55] were found in studies that reviewed medical charts (eight studies). Five studies combined a review of the medical chart with a self-report questionnaire to obtain a prevalence rate (range: 3.8–77.0%) [33, 66].

#### RMDs in general

In this category, 39 studies were included in which the overall prevalence rates varied from 0.6% [42] to 67.5% [21] with a median of 23.0%. Heterogeneity with regard to documentation of (symptoms of) RMDs was high: as an example, 14 definitions were found to describe RMDs in general (e.g., “arthritis”,”musculoskeletal disease”, and”osteoarticular problems”). Two studies used a database and found a prevalence rate between 2.0% [45] and 60.7% [47]. There was one study in which a physical examination of the foot was performed, they found a prevalence rate for foot conditions of 26.7% [50]. A self-report questionnaire was used in 11 studies reporting prevalence rates between 2.8% [27] and 67.5% [61]. Most studies reviewed the medical chart and showed prevalence rates which ranged from 0.6% [42] to 61.0% [29] (18 studies). Seven studies used a combination of a review of the medical chart and a self-report questionnaire (range of prevalence rates: 1.1–66.5%) [57, 66].

#### Specific type of RMD

Fourteen studies described prevalence rates of a specific type of RMD (gout 0.7–10.7%; median 1.8%, rheumatoid arthritis 3.3–11.0%; median 3.6% and osteo-arthritis 2.8–75.4%; median 21.1%) [30, 42, 48, 53, 54, 67]. We found two database studies, one reporting the prevalence of rheumatoid arthritis being 3.3% [48] and the second reporting a prevalence for gout of 10.7% [54]. Nine studies used a review of the medical chart to obtain prevalence rates (gout 0.7–1.8%, rheumatoid arthritis 3.6–11.0% and osteo-arthritis 2.8–75.4%) [18, 30, 42, 53]. Three studies performed a review of the medical chart and a self-report questionnaire and found prevalence rates for osteo-arthritis varying from 10.3% [60] to 47.5% [33]. Ten (18.9%) studies included patients with dementia or cognitive impairment [21, 22, 31, 33, 36, 42, 53, 54, 66, 67]. There seemed to be no clear relationship between the prevalence of gout, rheumatoid arthritis and osteo-arthritis and dementia.

### Assessment of *bias*

Supplementary Material S2 identifies that 26 (49.1%) of the studies reporting on RMDs met > 15 AXIS criteria and 27 studies met 10–15 criteria (50.9%). None of the studies was categorized into the two lowest AXIS risk of bias categories. The mean number of positive scores was 15 (range: 10–19 positive scores). Overall, the risk of bias was low to moderate, and negative scores were in most cases due to a lack of information about sample size justification and non-responders.

## Discussion

In this review, the overall prevalence rates of (symptoms of) RMDs varied to a great extent (range: 0.6–77.0%). The combination of large heterogeneity in populations studied but even more in case definition and approaches to document (symptoms of) RMDs (case ascertainment) result in broad prevalence rates. Most studies used medical charts or self-report questionnaires to obtain prevalence rates without verification with a physical examination. As a result, data about the nature and severity of RMD-related symptoms such as movement restriction, stiffness or swollen joints are currently not available in nursing home residents.

An important explanation for the broad prevalence rates of (symptoms of) RMDs found in our review might be the lack of consistency with regard to case definition. For instance, 14 different definitions were found to describe RMDs, such as “arthritis”,”musculoskeletal disease” or”osteoarticular problems”. Although the methodological quality of the individual studies is moderate to good (AXIS criteria 10–15 or > 15), the unclear case definition among the studies makes it impossible to compare or to create any form of hierarchy in the obtained prevalence rates. Furthermore, most of the studies reporting on musculoskeletal pain only described pain in a certain body part or structure. Not only does the unclear case definition complicate the understanding of the prevalence of specific RMDs, but it is also likely that the causes of musculoskeletal pain in this population are still ill understood.

Also, differences in case ascertainment between studies might explain the broad prevalence rates. Studies that reviewed the medical charts of nursing home residents often used standardized data sets. These data sets only assess a limited set of medical conditions. As an example, the Aged Care Assessment Program (ACAP) has a maximum of ten International Classification of Diseases and Related Health Problems (ICD) conditions that can be inserted. RMDs might not be prioritized in the selection of inserted ICD conditions leading to underreporting of RMDs.

Older people often underreport pain and other musculoskeletal symptoms, as these symptoms are seen as a normal part of aging [21]. Further, not all health-care workers are aware that nursing home residents may be in pain, especially in residents with cognitive impairment [10]. In studies among community-dwelling older people prevalence rates of self-reported RMDs are higher than in nursing home residents [37]. As this might be underreporting by nursing home residents, we found no clear differences in prevalence rates of (symptoms of) RMDs depending on the case ascertainment in this review. It is possible that there is underreporting in both self-report questionnaires and medical charts of nursing home residents.

In the study of Nguyen et al., it was found that nursing home residents with dementia had a slightly lower gout prevalence than residents without dementia [54]. Dementia has been reported to complicate the diagnosis of gout which can lead to underdiagnosing of gout in this population [54]. Husebo et al. previously reported on the importance of correct diagnosis and management of gout to reduce pain and potentially alleviate behavioral problems in older people with dementia [69]. Other studies have shown that gout is inversely correlated to dementia due to the potential neuroprotective effects of uric acid and the usage of non-steroidal anti-inflammatory drugs [70]. Although not a specific aim of our study, we found no unambiguous relationship between dementia and gout or other RMDs. The prevalence rates of rheumatoid arthritis (3.3–11.0%) appear to be high in this SLR compared to prevalence rates in community-dwelling elderly in Europe (0.2–0.4%) [71]. This is especially the case in the studies which include a self-report questionnaire. Perhaps a previous joint complaint or joint swelling was incorrectly attributed to rheumatoid arthritis by nursing home residents, health-care workers or informal caregivers.

A previously published systematic review by Smith et al. on musculoskeletal pain in nursing home residents included 24 studies and reported a prevalence rate of 30.2% [8]. This study reported additionally that musculoskeletal pain had a detrimental effect on quality of life of nursing home residents by restricting their mobility, social engagement and overall independence [8]. Higher dependence with activities of daily living has previously been shown to be associated with nursing home admission [12]. To our knowledge, there is currently no research about the nature and extent of symptoms of RMDs in nursing home residents. The only study that performed a physical examination aimed to focus on foot conditions but not on swollen and tender joints in general, range of motion or stiffness [50]. Further, the SLR of Smith et al. acknowledged that there is a lack of literature on strategies and interventions to address musculoskeletal pain in nursing home residents [8]. Improved diagnosis and management of RMDs in nursing home residents, in terms of providing appropriate pain relief, should be considered to improve quality of life.

Some limitations of the present study should be addressed. First, we cannot exclude language and availability bias because we applied language restrictions and not for all studies a full text could be obtained. Second, the majority of the studies were conducted in Europe and North America and geographical bias cannot be excluded. Socio-economic inequality and differences in receiving nursing home care could be responsible for heterogeneity in the prevalence of RMDs in nursing home residents. Third, due to large clinical and methodological heterogeneity of the included studies, we choose to describe the results narratively. No meta-analysis or creating hierarchy in prevalence rates could reliably be performed in this SLR which makes it difficult to compare the results of the smaller studies or to settle controversies of apparently conflicting RMD prevalence rates.

There are currently no studies in nursing home residents available that describe the results of a thorough physical examination of the complete musculoskeletal system. Studies that provide information on how nursing home residents themselves assess the severity of their joint complaints are also scarce. We recommend an observational study stratified by dementia (dementia as primary admission reason opposed to chronic illness as primary admission reason). The primary objective should be to investigate the prevalence of RMDs by performing a physical examination of the number of tender and (bony or synovial) swollen joints. To gain more insight into the impact of the joint complaints, information about the presence and severity of (joint) pain and of mobility limitations should be gathered. The findings of the physical examination should be compared with the diagnosis in the medical record to understand whether an accurate (differential) diagnosis for the joint complaints is recorded in the medical record.

## Conclusion

The overall prevalence of (symptoms of) RMDs varied to a great extent due to the unclear documentation and classification of RMDs. This makes it difficult to define which specific condition is present or to clearly distinguish musculoskeletal pain from other causes of chronic pain in nursing home residents. Establishing agreement on a useful and practical classification to ultimately increase identification of RMDs in the nursing home setting is necessary to prevent misunderstandings between physicians and increase comparability and generalizability of (research) findings. Future research into the nature and severity of RMD-related symptoms in nursing home residents may also contribute to a better identification and classification of RMDs.

## Supplementary Information

Below is the link to the electronic supplementary material.

Supplementary file1 (DOCX 20 kb)

Supplementary file2 (DOCX 37 kb)
