# Supplementary material for: Prevalence of rheumatic and musculoskeletal diseases (RMDs) in nursing home residents: a systematic literature review
Source: Eur Geriatr Med. 2024 Sep 25;15(5):1245–58. doi: 10.1007/s41999-024-01067-x (PMC11615105; doi:10.1007/s41999-024-01067-x)
Supplement: Supplementary file 1 — Supplementary file1 (DOCX 20 kb) [file 41999_2024_1067_MOESM1_ESM.docx]

**Prevalence of Rheumatic and Musculoskeletal Diseases (RMDs) in nursing home residents: A systematic literature review**

Shennah Austen, MD^a,b,d^, Iris Kamps, MD^a^, Annelies E.R.C.H. Boonen, MD, PhD^c,d^, Jos M.G.A. Schols, MD, PhD^b,d^, Marloes G.B. van Onna, MD, PhD^c,d^

^a^Cicero Zorggroep, Zuid-Limburg, The Netherlands, ^b^Department of Health Services Research, Maastricht University, Maastricht, The Netherlands, ^c^Department of Internal Medicine, division of Rheumatology, Maastricht University Medical Center, Maastricht, The Netherlands, ^d^School for Public Health and Primary Care (CAPHRI), Maastricht University, Maastricht, The Netherlands

**Corresponding Author:**

Shennah Austen, P.O. Box 149, 6440 AC, Brunssum, +31(0)455637400, s.austen@cicerozorggroep.nl

**Supplementary Material S1** Search strategies

**Pubmed/ MEDLINE**

("Residential Facilities"[Mesh] OR "Housing for the Elderly"[Mesh] OR "Long-Term Care"[Mesh] OR "Nursing Care"[Mesh])

OR

("home for the aged"[tiab] OR "old age home*"[tiab] OR "housing for the elder*"[tiab] OR "elderly hous*"[tiab] OR "long-term care"[tiab] OR "extended care"[tiab] OR "chronic care"[tiab] OR "nursing care"[tiab] OR "residential aged care"[tiab])

OR

(("long-term"[ti] OR "long-stay"[ti] OR nursing[ti] OR sheltered[ti] OR resident*[ti] OR retirement*[ti] OR care[ti] OR rest*[ti] OR skilled[ti] OR extented[ti] OR assisted[ti] OR group[ti]) AND (home*[ti] OR house*[ti] OR institution*[ti] OR facilit*[ti] OR center*[ti] OR centre*[ti] OR accommodation*[ti]))

OR

(("nursing home*"[tiab] OR "care home*"[tiab] OR "retirement home*"[tiab] OR "rest home*"[tiab] OR "care institution*" [tiab] OR "long term facilit*"[tiab] OR "nursing facilit*"[tiab] OR "skilled facilit*"[tiab] OR "extended facilit*"[tiab] OR "residential care cent*"[tiab] OR "assisted living facilit*"[tiab]) NOT ("nursing home*"[ti] OR "care home*"[ti] OR "retirement home*"[ti] OR "rest home*"[ti] OR "care institution*" [ti] OR "long term facilit*"[ti] OR "nursing facilit*"[ti] OR "skilled facilit*"[ti] OR "extended facilit*"[ti] OR "residential care cent*"[ti] OR "assisted living facilit*"[ti]))

("Musculoskeletal Diseases"[Mesh] OR ("Musculoskeletal System/abnormalities"[Mesh] OR "Musculoskeletal System/diagnosis"[Mesh] OR "Musculoskeletal System/epidemiology"[Mesh]))

OR

("musculoskeletal pain*"[tiab] OR "musculoskeletal disease*"[tiab] OR "orthopedic disorder*"[tiab] OR "musculoskeletal disorder*"[tiab] OR "musculoskeletal condition*"[tiab] OR "musculoskeletal symptom*"[tiab] OR arthros*[tiab] OR osteoarthr*[tiab] OR "degenerative joint disease*"[tiab] OR arthriti*[tiab] OR polyarthr*[tiab] OR rheuma*[tiab])

("Prevalence"[Mesh] OR "Epidemiology"[Mesh])

OR

(prevalen*[tiab] OR epidemiolog*[tiab] OR frequenc*[tiab] OR frequent*[tiab] OR morbidit*[tiab] OR occurrenc*[tiab] OR "epidemiology"[Subheading])

**CINAHL**

((MH "Residential Facilities+") OR (MH "Housing for Older Persons") OR (MH "Long Term Care") OR (MH "Nursing Care"))

OR

TI("home* W2 aged" OR "old age home*" OR "hous* W2 elder*" OR "elderly hous*" OR "long-term care" OR "extended care" OR "chronic care" OR "nursing care" OR "residential aged care")

OR

AB("home* W2 aged" OR "old age home*" OR "hous* W2 elder*" OR "elderly hous*" OR "long-term care" OR "extended care" OR "chronic care" OR "nursing care" OR "residential aged care")

OR

TI(("long-term" OR "long-stay" OR nursing OR sheltered OR resident* OR retirement* OR care OR rest* OR skilled OR extented OR assisted OR group) AND (home* OR house* OR institution* OR facilit* OR cent* OR accommodation*))

OR

AB("nursing home*" OR "care home*" OR "retirement home*" OR "rest home*" OR "care institution*" OR "long term facilit*" OR "nursing facilit*" OR "skilled facilit*" OR "extended facilit*" OR "residential care cent*" OR "assisted living facilit*")

(MH "Musculoskeletal Diseases+") OR (MH "Musculoskeletal System+/AB/PP")

OR

TI("musculoskeletal pain*" OR "musculoskeletal disease*" OR "orthopedic disorder*" OR "musculoskeletal disorder*" OR "musculoskeletal condition*" OR "musculoskeletal symptom*" OR arthros* OR osteoarthr* OR "degenerative joint disease*" OR arthriti* OR polyarthr* OR rheuma*)

OR

AB("musculoskeletal pain*" OR "musculoskeletal disease*" OR "orthopedic disorder*" OR "musculoskeletal disorder*" OR "musculoskeletal condition*" OR "musculoskeletal symptom*" OR arthros* OR osteoarthr* OR "degenerative joint disease*" OR arthriti* OR polyarthr* OR rheuma*)

((MH "Epidemiology") OR (MH "Comorbidity) OR (MH "Morbidity") OR (MH "Prevalence))

OR

TI (prevalen* OR epidemiolog* OR frequenc* OR frequent* OR morbidit* OR occurrenc*)

OR

AB (prevalen* OR epidemiolog* OR frequenc* OR frequent* OR morbidit* OR occurrenc*)

**Web of science**

TS=("home* for the aged" OR "old age home*" OR "hous* for the elder*" OR "elderly hous*" OR "long-term care" OR "extended care" OR "chronic care" OR "nursing care" OR "residential aged care")

OR

TI=(("long-term" OR "long-stay" OR nursing OR sheltered OR resident* OR retirement* OR care OR rest* OR skilled OR extented OR assisted OR group) AND (home* OR house* OR institution* OR facilit* OR cent* OR accommodation*))

OR

AB=("nursing home*" OR "care home*" OR "retirement home*" OR "rest home*" OR "care institution*" OR "long term facilit*" OR "nursing facilit*" OR "skilled facilit*" OR "extended facilit*" OR "residential care cent*" OR "assisted living facilit*")

TS=("musculoskeletal pain*" OR "musculoskeletal disease*" OR "orthopedic disorder*" OR "musculoskeletal disorder*" OR "musculoskeletal condition*" OR "musculoskeletal symptom*" OR arthros* OR osteoarthr* OR "degenerative joint disease*" OR arthriti* OR polyarthr* OR rheuma*)

TS=(prevalen* OR epidemiolog* OR frequenc* OR frequent* OR morbidit* OR occurrenc*)

**EMBASE**

(residential home/ OR home for the aged/ OR nursing home/ OR long term care/ OR nursing care/)

OR

("home* for the aged".kw. OR "old age home*".ti,ab,kw. OR "hous* for the elder*".kw. OR "elderly hous*".ti,ab,kw. OR "long-term care".ti,ab,kw. OR "extended care".ti,ab,kw. OR "chronic care".ti,ab,kw. OR "nursing care".ti,ab,kw. OR "residential aged care".ti,ab,kw.)

OR

(("long-term".ti. OR "long-stay".ti. OR nursing.ti. OR sheltered.ti. OR resident*.ti. OR retirement*.ti. OR care.ti. OR rest*.ti. OR skilled.ti. OR extented.ti. OR assisted.ti. OR group.ti.) AND (home*.ti. OR house*.ti. OR institution*.ti. OR facilit*.ti. OR center*.ti. OR centre*.ti . OR accommodation*.ti.))

OR

("nursing home*".ab,kw. OR "care home*".ab,kw. OR "retirement home*".ab,kw. OR "rest home*".ab,kw. OR "care institution*" .ab,kw. OR "long term facilit*".ab,kw. OR "nursing facilit*".ab,kw. OR "skilled facilit*".ab,kw. OR "extended facilit*".ab,kw. OR "residential care cent*".ab,kw. OR "assisted living facilit*".ab,kw.)

(musculoskeletal disease/ OR exp arthropathy/)

OR

("musculoskeletal pain*".ti,ab,kw. OR "musculoskeletal disease*".ti,ab,kw. OR "orthopedic disorder*".ti,ab,kw. OR "musculoskeletal disorder*".ti,ab,kw. OR "musculoskeletal condition*".ti,ab,kw. OR "musculoskeletal symptom*".ti,ab,kw. OR arthros*.ti,ab,kw. OR osteoarthr*.ti,ab,kw. OR "degenerative joint disease*".ti,ab,kw. OR arthriti*.ti,ab,kw. OR polyarthr*.ti,ab,kw. OR rheuma*.ti,ab,kw.)

prevalence/ or epidemiology/ or comorbidity/ or morbidity/

OR

(prevalen*.ti,ab,kw. OR epidemiolog*.ti,ab,kw. OR frequenc*.ti,ab,kw. OR frequent*.ti,ab,kw. OR morbidit*.ti,ab,kw. OR occurrenc*.ti,ab,kw.)
