# Supplementary material for: Prevalence of rheumatic and musculoskeletal diseases (RMDs) in nursing home residents: a systematic literature review
Source: Eur Geriatr Med. 2024 Sep 25;15(5):1245–58. doi: 10.1007/s41999-024-01067-x (PMC11615105; doi:10.1007/s41999-024-01067-x)
Supplement: Supplementary file 2 — Supplementary file2 (DOCX 37 kb) [file 41999_2024_1067_MOESM2_ESM.docx]

**Prevalence of Rheumatic and Musculoskeletal Diseases (RMDs) in nursing home residents: A systematic literature review**

Shennah Austen, MD^a,b,d^, Iris Kamps, MD^a^, Annelies E.R.C.H. Boonen, MD, PhD^c,d^, Jos M.G.A. Schols, MD, PhD^b,d^, Marloes G.B. van Onna, MD, PhD^c,d^

^a^Cicero Zorggroep, Zuid-Limburg, The Netherlands, ^b^Department of Health Services Research, Maastricht University, Maastricht, The Netherlands, ^c^Department of Internal Medicine, division of Rheumatology, Maastricht University Medical Center, Maastricht, The Netherlands, ^d^School for Public Health and Primary Care (CAPHRI), Maastricht University, Maastricht, The Netherlands

**Corresponding Author:**

Shennah Austen, P.O. Box 149, 6440 AC, Brunssum, +31(0)455637400, s.austen@cicerozorggroep.nl

| **Supplementary Material S2** Risk of bias assessment with AXIS tool | | | | | | | | | | | | | | | | | | | | | |
| --- | --- | --- | --- | --- | --- | --- | --- | --- | --- | --- | --- | --- | --- | --- | --- | --- | --- | --- | --- | --- | --- |
| **Author, publication year** | **ROB-1** | **ROB-2** | **ROB-3** | **ROB-4** | **ROB-5** | **ROB-6** | **ROB-7** | **ROB-8** | **ROB-9** | **ROB-10** | **ROB-11** | **ROB-12** | **ROB-13*** | **ROB-14** | **ROB-15** | **ROB-16** | **ROB-17** | **ROB-18** | **ROB-19*** | **ROB-20** | **Score** |
| Abell, 2004 [21] | Yes | Yes | Yes | Yes | ? | Yes | No | Yes | Yes | No | No | No | No | No | Yes | No | Yes | Yes | ? | ? | 11/20 |
| Achterberg, 2007 [22] | Yes | Yes | No | Yes | Yes | Yes | Yes | Yes | Yes | Yes | Yes | Yes | No | Yes | Yes | Yes | Yes | Yes | ? | Yes | 18/20 |
| Alaba, 2009 [23] | Yes | Yes | No | Yes | Yes | ? | ? | Yes | Yes | No | No | Yes | ? | No | Yes | Yes | Yes | No | No | ? | 11/20 |
| Al-Momani, 2016 [24] | Yes | Yes | No | Yes | No | No | Yes | Yes | Yes | Yes | Yes | Yes | Yes | Yes | No | Yes | No | Yes | No | Yes | 14/20 |
| Albertsen, 2021 [25] | Yes | Yes | No | Yes | Yes | Yes | Yes | Yes | Yes | Yes | No | Yes | No | Yes | Yes | Yes | Yes | Yes | No | Yes | 18/20 |
| Algameel, 2020 [26] | Yes | Yes | No | Yes | Yes | Yes | No | No | No | Yes | Yes | Yes | No | No | Yes | Yes | Yes | Yes | No | Yes | 15/20 |
| Altiparmak, 2011 [27] | Yes | Yes | Yes | Yes | Yes | No | No | ? | No | Yes | No | Yes | ? | No | Yes | Yes | Yes | No | No | Yes | 12/20 |
| Bekhet, 2014 [28] | Yes | Yes | Yes | Yes | Yes | No | ? | Yes | Yes | Yes | Yes | Yes | No | No | No | Yes | No | No | ? | Yes | 13/20 |
| Black, 2006 [29] | Yes | Yes | No | Yes | Yes | Yes | Yes | Yes | Yes | Yes | Yes | Yes | No | Yes | Yes | Yes | Yes | Yes | No | Yes | 19/20 |
| Boerlage, 2008 [10] | Yes | Yes | No | Yes | Yes | No | ? | Yes | Yes | Yes | Yes | Yes | No | Yes | Yes | Yes | Yes | Yes | No | Yes | 17/20 |
| Chen, 2023 [64] | Yes | Yes | Yes | Yes | Yes | Yes | ? | Yes | Yes | Yes | Yes | Yes | ? | No | Yes | Yes | Yes | Yes | No | Yes | 17/20 |
| Cook, 1998 [30] | Yes | Yes | Yes | Yes | Yes | Yes | Yes | Yes | Yes | No | No | Yes | Yes | Yes | Yes | Yes | Yes | Yes | ? | Yes | 16/20 |
| D’Astolfo, 2006 [31] | Yes | Yes | No | Yes | Yes | Yes | Yes | Yes | Yes | No | Yes | Yes | No | Yes | Yes | Yes | Yes | Yes | No | Yes | 18/20 |
| Damian, 2004 [32] | Yes | Yes | Yes | Yes | Yes | Yes | ? | Yes | Yes | No | Yes | Yes | No | Yes | Yes | Yes | Yes | Yes | ? | ? | 16/20 |
| Decker, 2009 [33] | Yes | Yes | Yes | Yes | Yes | Yes | Yes | Yes | Yes | Yes | Yes | Yes | No | Yes | Yes | Yes | Yes | Yes | ? | Yes | 19/20 |
| Ferrell, 1990 [34] | Yes | Yes | No | Yes | Yes | Yes | Yes | Yes | No | Yes | Yes | Yes | No | Yes | Yes | Yes | Yes | Yes | ? | ? | 16/20 |
| Finne-Soveri, 2000 [35] | Yes | Yes | No | Yes | Yes | Yes | Yes | Yes | Yes | No | Yes | Yes | No | No | No | Yes | Yes | No | No | ? | 14/20 |
| Fisher, 2002 [36] | Yes | Yes | No | Yes | Yes | No | No | Yes | Yes | Yes | Yes | Yes | No | No | Yes | Yes | Yes | Yes | No | ? | 15/20 |
| Gill,  2022 [37] | Yes | Yes | No | Yes | Yes | Yes | ? | Yes | Yes | Yes | Yes | Yes | ? | No | Yes | Yes | Yes | Yes | No | Yes | 16/20 |
| Gerber, 2016 [38] | Yes | Yes | No | Yes | Yes | Yes | ? | Yes | Yes | No | Yes | Yes | ? | No | Yes | Yes | Yes | Yes | ? | Yes | 14/20 |
| Grimby, 1999 [39] | No | Yes | No | Yes | ? | ? | Yes | Yes | No | No | No | Yes | Yes | Yes | Yes | Yes | Yes | Yes | Yes | Yes | 11/20 |
| Guccione, 1989 [40] | Yes | Yes | No | Yes | Yes | Yes | No | Yes | Yes | Yes | Yes | Yes | ? | No | Yes | Yes | Yes | Yes | No | ? | 15/20 |
| Hillen, 2017 [41] | Yes | Yes | No | Yes | Yes | Yes | No | Yes | Yes | No | Yes | Yes | No | No | Yes | Yes | Yes | Yes | No | Yes | 17/20 |
| Hsieh, 2021 [42] | Yes | Yes | No | Yes | Yes | ? | ? | Yes | Yes | Yes | Yes | Yes | Yes | No | Yes | Yes | Yes | Yes | No | Yes | 16/20 |
| Jerez-Roig, 2013 [43] | Yes | Yes | No | Yes | Yes | ? | ? | Yes | Yes | Yes | Yes | Yes | Yes | Yes | Yes | Yes | Yes | Yes | No | Yes | 16/20 |
| Kalideen, 2022 [44] | Yes | Yes | No | Yes | Yes | No | No | Yes | Yes | Yes | Yes | Yes | Yes | No | Yes | Yes | Yes | Yes | No | Yes | 15/20 |
| Karmel, 2012 [45] | Yes | Yes | No | Yes | Yes | Yes | ? | Yes | Yes | No | Yes | Yes | No | No | Yes | Yes | Yes | Yes | ? | ? | 14/20 |
| Laikhuram, 2024 [65] | Yes | Yes | Yes | Yes | Yes | Yes | ? | Yes | Yes | Yes | Yes | Yes | ? | No | Yes | Yes | Yes | Yes | No | Yes | 17/20 |
| Lapane, 2012 [46] | Yes | Yes | No | Yes | Yes | Yes | ? | Yes | Yes | Yes | Yes | Yes | No | No | Yes | Yes | Yes | Yes | Yes | Yes | 16/20 |
| Lind, 2020 [47] | Yes | Yes | No | Yes | Yes | Yes | ? | Yes | Yes | Yes | Yes | Yes | ? | No | Yes | Yes | Yes | Yes | Yes | Yes | 15/20 |
| Luque Ramos, 2017 [48] | Yes | Yes | No | Yes | Yes | Yes | ? | Yes | Yes | No | No | Yes | ? | No | Yes | Yes | Yes | Yes | Yes | No | 12/20 |
| Marques, 2015 [49] | Yes | Yes | No | Yes | Yes | Yes | Yes | Yes | Yes | Yes | Yes | Yes | No | Yes | Yes | Yes | No | No | No | Yes | 17/20 |
| Martinez, 2011 [50] | Yes | Yes | No | No | Yes | ? | ? | Yes | Yes | Yes | No | No | ? | Yes | Yes | No | Yes | Yes | No | ? | 11/20 |
| Monroe, 2011 [51] | Yes | Yes | No | Yes | Yes | Yes | ? | Yes | Yes | No | Yes | Yes | No | Yes | Yes | Yes | Yes | Yes | ? | Yes | 16/20 |
| Moore, 2012 [52] | Yes | Yes | No | Yes | Yes | Yes | ? | Yes | Yes | No | Yes | Yes | ? | No | Yes | Yes | Yes | Yes | No | No | 15/20 |
| Myrenget, 2023 [66] | Yes | Yes | No | Yes | No | No | ? | Yes | Yes | Yes | Yes | Yes | ? | No | Yes | Yes | Yes | Yes | No | Yes | 14/20 |
| Ng,  2020 [53] | Yes | Yes | No | Yes | ? | ? | ? | Yes | Yes | No | No | Yes | ? | No | Yes | Yes | Yes | No | No | No | 10/20 |
| Nguyen, 2020 [54] | Yes | Yes | No | Yes | Yes | Yes | ? | Yes | Yes | Yes | Yes | Yes | ? | No | Yes | Yes | Yes | Yes | No | Yes | 16/20 |
| Peng, 2009 [55] | Yes | Yes | No | Yes | Yes | ? | ? | Yes | Yes | Yes | Yes | Yes | ? | No | Yes | Yes | Yes | Yes | No | Yes | 15/20 |
| Proctor, 200 [56] | No | Yes | No | Yes | Yes | ? | ? | Yes | Yes | No | Yes | Yes | ? | No | Yes | Yes | Yes | No | ? | No | 10/20 |
| v Rensbergen, 2010 [15] | Yes | Yes | Yes | Yes | Yes | Yes | ? | Yes | Yes | ? | Yes | Yes | ? | No | Yes | Yes | Yes | Yes | ? | ? | 14/20 |
| Sawyer, 2007 [9] | Yes | Yes | No | Yes | Yes | Yes | Yes | Yes | Yes | Yes | Yes | Yes | No | Yes | Yes | Yes | Yes | Yes | ? | Yes | 18/20 |
| Sigurdardottir, 2018 [57] | Yes | Yes | No | Yes | Yes | Yes | Yes | Yes | Yes | Yes | Yes | Yes | No | Yes | Yes | Yes | Yes | Yes | No | Yes | 19/20 |
| Takai, 2013 [58] | Yes | Yes | No | Yes | Yes | Yes | No | Yes | No | No | Yes | Yes | Yes | No | Yes | Yes | Yes | Yes | ? | Yes | 13/20 |
| Tansug, 2021 [14] | Yes | Yes | Yes | Yes | No | No | ? | Yes | Yes | Yes | Yes | Yes | ? | No | Yes | Yes | Yes | Yes | No | Yes | 15/20 |
| Torvik, 2009 [59] | Yes | Yes | Yes | Yes | Yes | Yes | ? | Yes | Yes | Yes | Yes | Yes | No | Yes | Yes | Yes | Yes | Yes | ? | Yes | 17/20 |
| Tsai,  2004 [60] | Yes | Yes | No | Yes | Yes | Yes | Yes | Yes | Yes | No | Yes | Yes | No | Yes | Yes | Yes | Yes | Yes | ? | Yes | 17/20 |
| Tse,  2004 [61] | Yes | Yes | No | Yes | Yes | Yes | No | Yes | ? | No | No | Yes | ? | No | Yes | No | Yes | Yes | No | Yes | 12/20 |
| Veal, 2019 [62] | Yes | Yes | No | Yes | Yes | Yes | Yes | Yes | Yes | No | Yes | Yes | No | Yes | Yes | Yes | Yes | Yes | No | Yes | 18/20 |
| Vetrano, 2022 [67] | Yes | Yes | No | No | ? | ? | ? | Yes | Yes | Yes | Yes | Yes | ? | No | Yes | Yes | Yes | Yes | No | Yes | 12/20 |
| Xie, 2023 [68] | Yes | Yes | Yes | Yes | Yes | Yes | ? | Yes | Yes | Yes | Yes | Yes | ? | No | Yes | Yes | Yes | Yes | No | Yes | 17/20 |
| Zanocchi, 2008 [63] | Yes | Yes | No | Yes | Yes | No | Yes | Yes | Yes | No | No | No | Yes | Yes | Yes | Yes | Yes | Yes | ? | Yes | 13/20 |
| Zarowitz, 2013 [18] | Yes | Yes | No | Yes | Yes | Yes | No | Yes | Yes | No | Yes | Yes | No | Yes | Yes | Yes | Yes | Yes | No | No | 17/20 |

*ROB = Risk of bias; ? = Unknown (an unknown is a negative score). *Reverse coding required for this question (therefore a no is a positive score). ROB-1 = Were the aims/objectives of the study clear? ROB-2 = Was the study design appropriate for the stated aim(s)? ROB-3 = Was the sample size justified? ROB-4 = Was the target/reference population clearly defined? ROB-5 = Was the sample frame taken from an appropriate population base so that it is closely represented the target/reference population under investigation? ROB-6 = Was the selection process likely to select subjects/participants that were representative of the target/reference population under investigation? ROB-7 = Were measures undertaken to address and categorize non-responders? ROB-8 = Were the risk factors and outcome variables measured appropriate to the aims of the study? ROB-9 = Were the risk factors and outcome variables measured correctly using instruments/ measurements that had been trialled, piloted or published previously? ROB-10 = Is it clear what was used to determined statistical significance and/or precision estimates? (e.g., p values, CIs) ROB-11 = Were the methods (including statistical methods) sufficiently described to enable them to be repeated? ROB-12 = Were the basic data adequately described? ROB-13 = Does the response rate raise concerns about nonresponse bias? ROB-14 = If appropriate, was information about non-responders described? ROB-15 = Were the results internally consistent? ROB-16 = Were the results for the analyses described in the methods, presented? ROB-17 = Were the authors’ discussions and conclusions justified by the results? ROB-18 = Were the limitations of the study discussed? ROB-19 = Were there any funding sources or conflicts of interest that may affect the authors’ interpretation of the results? ROB-20 = Was ethical approval or consent of participants attained?*
